# Diagnostic performance of *Mycoplasmopsis bovis* antibody ELISA tests on bulk tank milk from dairy herds

**DOI:** 10.1186/s12917-024-03927-x

**Published:** 2024-03-05

**Authors:** Jade Bokma, Martin Kaske, Jeroen Vermijlen, Sabrina Stuyvaert, Bart Pardon

**Affiliations:** 1 Department of Internal Medicine, Reproduction, and Population Medicine, Faculty of Veterinary Medicine, Ghent University, Merelbeke, Belgium; 2 Swiss Bovine Health Service, Zurich, Switzerland; 3 Rubovet, Ploegsteert, Belgium

**Keywords:** Bayesian latent class analysis, Bio K302, Bio K432, Cutoff, ID-Screen, Screening

## Abstract

**Background:**

Testing of bulk tank milk (BTM) for *Mycoplasmopsis bovis* (previously *Mycoplasma bovis*) antibodies is increasingly popular. However the performance of some commercially available tests is unknown, and cutoff values possibly need to be adjusted in light of the purpose. Therefore, the aim of this study was to compare the diagnostic performance of three commercially available *M. bovis* antibody ELISAs on BTM, and to explore optimal cutoff values for screening purposes. A prospective diagnostic test accuracy study was performed on 156 BTM samples from Belgian and Swiss dairy farms using Bayesian Latent Class Analysis. Samples were initially classified using manufacturer cutoff values, followed by generated values.

**Results:**

Following the manufacturer’s guidelines, sensitivity of 91.4%, 25.6%, 69.2%, and specificity of 67.2%, 96.8%, 85.8% were observed for ID-screen, Bio K432, and Bio K302, respectively. Optimization of cutoffs resulted in a sensitivity of 89.0%, 82.0%, and 85.5%, and a specificity of 83.4%, 75.1%, 77.2%, respectively.

**Conclusions:**

The ID-screen showed the highest diagnostic performance after optimization of cutoff values, and could be useful for screening. Both Bio-X tests may be of value for diagnostic or confirmation purposes due to their high specificity.

**Supplementary Information:**

The online version contains supplementary material available at 10.1186/s12917-024-03927-x.

## Background

*Mycoplasmopsis bovis* (previously *Mycoplasma bovis*) is a small bacterium, causing huge economic losses, hampered animal welfare, and high antimicrobial use due to pneumonia, otitis, arthritis, and mastitis [1–3]. As *M. bovis* demonstrates both inherent and acquired resistance against many antimicrobials and no proven effective vaccine is available, the control of *M. bovis* is very challenging. Emphasis should be on the prevention of *M. bovis* entering the herd or to limit its spread through the herd as soon as possible. The most identified risk factor for introduction of *M. bovis* into the herd is purchase [4, 5], while transmission within the herd can be continued by direct contact, calves drinking infected milk, and housing-related factors such as the absence of an individual calving pen or overcrowding [5–9]. When purchasing animals, screening of individual animals by antigen and antibody detection has been proposed. However, tests are imperfect, and intermittent shedding may prevent the identification of carrier animals [10]. Knowledge about herd status of animals can contribute to a reduced risk of introducing *M. bovis* into new herds, and allows to monitor the effect of treatment or management implementations. One way to easily screen dairy farms is by monitoring the bulk tank milk (BTM) for antibodies (e.g. ELISA) or antigen (e.g. PCR, culture) [11, 12]. As milk from mastitis cows is often withhold from the BTM, antibody ELISA is preferred over PCR in national programs [6, 13, 14]. Nevertheless, interpretation of antibody ELISA test results can be challenging due to performance variability of tests, inter-laboratory variation, mutable cutoff values, and the target population [15–18]. Therefore, commercially available tests are often favored. So far, many studies compared commercially available antibody ELISAs showing superiority of the new ID-screen *Mycoplasma bovis* indirect (ID-Vet, Grabels, France) over Bio-X tests (Bio-X Diagnostics, Rochefort, Belgium) on serum [15, 17, 19]. This test was subsequently adopted to determine the prevalence of *M. bovis* in several countries [19–21]. However, so far the diagnostic performance of the ID-screen has not been reported for BTM samples, and only one study investigated the use of a commercially available ELISA on BTM samples (the Bio K302) [16]. As the sensitivity and specificity of such tests in different populations can have a great impact on the applicability of the test for different purposes (e.g. screening, diagnosis) and interpretation for follow-up measures, the objective of this study was (1) to compare diagnostic test accuracy of three commercial antibody ELISAs for *M. bovis* (ID-screen, Bio K302, Bio K432) on BTM from Belgian and Swiss dairy herds using Bayesian Latent Class Analysis (BLCA), and (2)  to explore the optimal cutoff values for all three antibody ELISA tests as a screening tool for *M. bovis* antibodies in BTM.

## Results

### Study population and antibody prevalence

When using manufacturer cutoffs, out of the 156 BTM samples, 30.8% (48/156) tested positive for *M. bovis* antibodies in the BTM using Bio K302_≥ 37%_, 9.6% (15/156) using Bio K432_≥ 40%_, and 50.6% (79/156) using ID-screen_≥ 30%_. When categorizing results of the ID-screen the total number of positive BTM samples (both Belgian and Swiss herds) was 50.6% (79/156, CO_≥ 30%_), 35.3% (55/156, CO_≥ 50%_), 18.6% (29/156, CO_≥ 100%_), and 5.8% (9/156, CO_≥ 150%_). For Bio K302 this was 21.2% (33/156, CO_≥ 50%_), 28.9% (45/156, CO_≥ 40%_), 39.1% (61/156, CO_≥ 30%_), 67.3% (105/156, CO_≥ 20%_), and 91.7% (143/156, CO_≥ 10%_), while this was 5.8% (9/156, CO_≥ 50%_), 17.3% (27/156, CO_≥ 30%_), 39.7% (62/156, CO_≥ 20%_), and 73.7% (115/156, CO_≥ 10%_) for the Bio K432 (Supplementary File 1). Out of the 85 BTM samples from Belgian dairy herds, 38.8% (33/85) tested positive for *M. bovis* antibodies in the BTM using Bio K302_≥ 37%_, 14.1% (12/85) using Bio K432_≥ 40%_, and 61.2% (52/85) using ID-screen_≥ 30%_. For Swiss herds this was 21.1% (15/71), 4.2% (3/71), and 38.0% (27/71).

### Bayesian latent class analysis

First, the ID-screen, Bio K432, and Bio K302 were compared using cutoff values proposed by the manufacturer. Both conditional dependent and conditional independent models were built for three different priors. All results are shown in Table 1, except dependent model 1 and 2, due to a lack of convergence. The independent non informative model (independent model 1) was used as model for further Bayesian latent class analysis (Table 1, bold) for the following reasons: (1) the third model (both independent and dependent) had a higher (38.49–39.68) DIC than model 1 (37.34) and 2 (37.05), (2) the third model showed for both the independent and dependent model some variation (10–15%) for ID-screen sensitivity and K302 specificity in comparison to model 1 and 2, and (3) the sensitivity analysis showed great influence of adding extreme prior information on sensitivity, specificity, and prevalence. However, the used prior information, may not be completely representative for the aim of this study, as in the study of Nielsen et al. (2015), the latent class could have been different due to comparison with PCR (detection of antigen) instead of antibodies. Also the true prevalence of *M. bovis* antibodies in our study population could have changed greatly over time. Independent model 1 showed a high sensitivity for ID-screen (91.4%), a low sensitivity for Bio K432 (25.6%) and a moderate sensitivity for Bio K302 (69.2%). The specificity was moderate for the ID-screen (67.2%), while high for Bio K432 (96.8%) and Bio K302 (85.8%). Credible intervals are shown in Table 1.

**Table 1:** Posterior median, 95% credible interval (CI95), and the deviance information criterion (DIC) for three conditional independent models and one conditional dependent model for the sensitivity (Se), specificity (Sp), and prevalence (prev) of three *Mycoplasma bovis* antibody ELISA tests (ID screen, Bio K302, Bio K432) on 156 bulk tank milk samples from Belgian (*n* = 85) and Swiss (*n* = 71) dairy herds. Manufacturer cutoff values for the sample-to-positive percentage (S/P%) were used. The first independent model (bold) was used for further Bayesian latent class modelling

		INDEPENDENT			DEPENDENT
Test_cutoff(S/P%)_	Parameter	Model 1^a^	Model 2^b^	Model 3^c^	Model 3^c^
ID_≥ 30%_	Se (%) (CI95)	**91.4 (70.7–99.7)**	91.0 (70.7–99.6)	78.8 (64.4–92.7)	77.4 (54.1–93.5)
ID_≥ 30%_	Sp (%) (CI95)	**67.2 (53.6–92.6)**	68.3 (54.7–92.6)	71.9 (57.8–93.3)	69.3 (51.5–93.1)
K432_≥ 40%_	Se (%) (CI95)	**25.6 (12.4–50.5)**	24.7 12.3–46.3)	19.5 (10.5–32.4)	19.3 (10.1–32.6)
K432_≥ 40%_	Sp (%) (CI95)	**96.8 (91.1–99.7)**	96.9 (91.2–99.7)	97.2 (90.9–99.8)	96.9 (90.3–99.8)
K302_≥ 37%_	Se (%) (CI95)	**69.2 (43.3–96.4)**	67.4 (43.2–95.2)	63.2 (45.8–81.4)	63.4 (45.8–82.0)
K302_≥ 37%_	Sp (%) (CI95)	**85.8 (74.8–97.4)**	86.3 (75.6–97.5)	96.4 (92.4–98.7)	96.4 (92.3–98.8)
	Prev (%) (CI95)	**30.8 (14.4–56.1)**	32.7 (17.4–55.0)	44.1 (30.0-61.9)	43.7 (29.4–62.1)
	covDn (CI95)	-	-	-	0.00 (-0.02-0.03)
	covDp (CI95)	-	-	-	0.01 (-0.06-0.13)
	DIC model	37.34	37.05	38.49	39.68

^a^Model 1: no informative priors

^b^Model 2: informative prior on prevalence of *M. bovis* in Flanders and Switzerland (mode, 40%; 95th percentile 16%) resulting in prior density Beta(3.223,4.335)^6,27,28^

^c^Model 3: informative prior on prevalence and Bio K302 sensitivity (mode 60.4%, 95th percentile 37.5%) and specificity (mode 97.3%, 95th percentile 94%) resulting in prior density Beta(8.086, 5.646) and Beta(147.175,5.056), respectively^16^

Secondly the different manufacturer S/P% cutoff values for the categorisation of ID-screen results (ID_≤ 30%_, ID_≤ 50%_, ID_≤ 100%,_ ID_≤ 150%_) were one by one compared in the BLCA to Bio K432_≤ 40%_ and Bio K302_≤ 37%_ results. When increasing the cutoff value of the ID-screen, the BLCA showed an increase in specificity (range 67.2-93.1%), with a slight improvement of sensitivity (91.6%) or decline (78.7%) (Table 2). The model had a lot of difficulties to converge when cutoff ≤ 150% was used, resulting in very broad CI95 intervals (Table 2) – probably due to the low number of positive samples for ID-screen. Using the S/P% cutoff of ≥ 50% and ≥ 100% resulted in the highest Youden index (J = 0.72), with a sensitivity of 91.6% for ID-screen_≤ 50_ and 78.7% for ID-screen_≤ 100_, whereas a specificity of 80.5% and 93.1%, were obtained, respectively. As a screening test is supposed to have the highest sensitivity possible, further analysis were performed with a cutoff value of ≥ 50% (Table 2, bold). Sensitivity and specificity including 95% credible intervals are shown in Table 2 for the three tests using the four different S/P% cutoff values.

**Table 2:** Posterior median and 95% credible interval (CI95) for a conditional independent test for the determination of sensitivity (Se) and specificity (Sp) of three *Mycoplasma bovis* antibody ELISA tests on 156 bulk tank milk from dairy herds while using four different cutoff values for the sample-to-positive percentage (S/P%) of the ID-screen. The optimal cutoff value of ≥ 50% for the ID-screen (bold) was used for further Bayesian latent class modelling

Test _cutoff (S/P%)_	ID-screen_≥ 30%_		ID-screen_≥ 50%_		ID-screen_≥ 100%_		ID-screen_≥ 150%_ *	
	Se (%) (CI95)	Sp (%) (CI95)	Se (%) (CI95)	Sp (%) (CI95)	Se (%) (CI95)	Sp (%) (CI95)	Se (%) (CI95)	Sp (%) (CI95)
ID-screen^a^	91.4 (70.7–99.7)	67.2 (53.6–92.6)	**91.6 (66.5–99.7)**	**80.5 (68.5–96.7)**	78.7 (46.6–98.6)	93.1 (84.6–99.4)	13.7 (0.32–82.5)	93.8 (18.9–99.7)
Bio K432_≥ 40%_^b^	25.6 (12.4–50.5)	96.8 (91.1–99.7)	37.4 (18.2–74.5)	97.7 (93.0-99.8)	45.2 (22.5–82.6)	97.0 (92.1–99.7)	21.9 (0.85–91.1)	90.0 (9.46–99.2)
Bio K302_≥ 37%_^c^	69.2 (43.3–96.4)	85.8 (74.8–97.4)	66.8 (43.2–92.0)	79.3 (69.9–88.6)	69.0 (45.2–92.3)	76.8 (68.1–86.0)	44.1 (15.8–95.5)	66.7 (0.48–84.8)
Prev	30.8 (14.4–56.1)		22.2 (9.71–41.3)		16.8 (7.30–31.9)		28.9 (3.7–96.2)	

^a^ The following cut-off values for ID-screen were used: ≥30%, ≥ 50%, ≥ 100%, and ≥150, ^b^ Cut-off value for Bio K 432: ≥40%, ^c^ Cut-off value for Bio K 302: ≥37%

* Results for cutoff value of ≥ 150% were obtained by using ‘generation inits’ and resulted in very broad, not useful, CI95

Third, we explored different S/P% cutoff values to increase the moderate sensitivity of Bio K302 (69.2%), and low sensitivity of Bio K432 (25.6%). The highest Youden index (J = 0.54) was reached for Bio K302 when the cutoff value was set at ≥ 30%, with a sensitivity of 76.7% and specificity of 77.5% (Table 3, bold). For the Bio K432 the optimal cutoff value (J = 0.65) was set at ≥ 20%, with a sensitivity of 89.8% and specificity of 74.7% (Table 3, bold). Sensitivity and specificity including 95% credible intervals are shown in Table 3. The model for Bio K432 ≥ 10% was unidentifiable, but as an extra control, the cutoff of ≥ 10% was run in the final model. This resulted in a very low specificity (35.8%), and was therefore withhold from the final model.

**Table 3:** Posterior median and 95% credible interval (CI95) for a conditional independent test for the determination of sensitivity (Se), specificity (Sp), and prevalence (Prev) of three *Mycoplasma bovis* antibody ELISA tests (ID-screen≥ 50%, Bio K302, Bio K432) on 156 bulk tank milk samples from Belgian and Swiss dairy herds, while using different cutoff values for the sample-to-positive percentage (S/P%) of the Bio K302 and Bio K432. Optimal cutoff values and results for Bio K302 and Bio K432 are highlighted bold

Test	S/P% cutoff	Se (%) (CI95)	Sp (%) (CI95)	Prev (%) (CI95)	Youden’s index (J)
Bio K302	≥ 50%	59.6 (34.7–88.2)	88.1 (80.2–95.3)	20.1 (9.0-38.7)	0.48
	≥ 40%	67.0 (43.6–92.1)	82.5 (73.4–91.6)	23.4 (11.1–41.8)	0.50
	≥ 37%	66.8 (43.2–92.0)	79.3 (69.9–88.6)	22.2 (9.71–41.3)	0.46
	**≥ 30%**	**76.7 (58.2–94.7)**	**77.5 (67.1–90.3)**	**30.9 (18.1–47.6)**	**0.54**
	≥ 20%*	58.6 (45.5–68.8)	13.5 (2.6–28.7)	69.5 (50.2–88.6)	-0.28
Bio K432	≥ 50%	74.8 (47.0-96.1)	99.3 (96.2–100.0)	19.3 (8.0-37.8)	0.29
	≥ 40%	37.4 (18.2–74.5)	97.7 (93.0-99.8)	22.2 (9.7–41.3)	0.35
	≥ 30%	65.3 (37.6–95.3)	93.3 (87.0-98.1)	18.7 (9.7–34.6)	0.59
	**≥ 20%**	**89.8 (67.2–99.5)**	**74.7 (64.9–84.9)**	**22.5 (13.2–37.6)**	**0.65**
	≥ 10%**	-	-	-	-

* Results for BIO K302 cutoff value of ≥ 20% were obtained by using ‘generation inits’

** Model unidentifiable

The final model, including ID-screen_≥ 50%_, Bio K302_≥ 30%_ and Bio K432_≥ 20%,_ shows the highest sensitivity for ID-screen (89.0%), followed by Bio K302 (85.5%), and Bio K432 (82.0%). The specificity is following the same order, being 83.4% for ID-screen, 77.2% for Bio K302, and 75.1% for Bio K432. The 95% credible intervals are shown in Table 4, reflecting no significant difference in diagnostic tests accuracy. Though, highest Youdens index is obtained for the ID-screen_≥ 50_ (J = 0.72). The sensitivity analysis showed the final model to be robust to changes in the prior distribution, only when extreme low values for sensitivity with high certainty were included as prior, a deviation from the 95% CI of the final model was observed.

**Table 4:** Posterior median and 95% credible interval (CI95) for the conditional independent test for the determination of sensitivity (Se), specificity (Sp), and prevalence (Prev) of three *Mycoplasma bovis* antibody ELISA tests (ID screen, Bio K302, Bio K432) on 156 bulk tank milk samples from Belgian and Swiss dairy herds while using optimized cutoff values for the sample-to-positive percentage (S/P%) (final model)

Test	S/P% cutoff	Se (%) (CI95)	Sp (%) (CI95)	Prev (%) (CI95)	Youden index (J)
ID-screen	≥ 50%	89.0 (67.7–99.4)	83.4 (73.7–93.5)	26.1 (16.3–40.5)	0.72
Bio K302	≥ 30%	85.5 (64.9–98.9)	77.2 (67.6–86.6)	26.1 (16.3–40.5)	0.63
Bio K432	≥ 20%	82.0 (62.0-97.7)	75.1 (65.7–84.3)	26.1 (16.3–40.5)	0.57

## Discussion

In this study we had two objectives. First, we sought to assess the performance of *M. bovis* antibody ELISA tests (Bio K302, Bio K432, and ID-screen) on BTM samples from Belgian and Swiss dairy herds. Secondly, we explored the optimal cutoff values for utilizing these tests as a screening tool, therefore maximizing sensitivity to prevent the misclassification of false negative herds. We performed a BLCA to determine the performance of the three different tests. As this kind of analysis searches for common ground between tests (the latent class), this was in all probability the presence of antibodies against *M. bovis*. Nevertheless, interpretation of diagnostic performance results should be taken prudently. Also, there is limited knowledge regarding the association with clinical status for some of the antibody ELISAs, and the duration of *M. bovis* antibodies after exposure. The detection of antibodies in BTM could therefore reflect an infected herd, but also a non-infectious herd with immunity. Keeping this in mind, our study yielded several noteworthy observations.

For the ID-screen, the model following manufacturer recommendations (cutoff ≥ 30%) showed a high sensitivity (91.4%), but rather low specificity (67.2%). When enhancing the cutoff to ≥ 50% we observed a fair increase in specificity (83.4%). Such an influence was also observed for the ID-screen when used on serum samples from youngstock [17]. When specificity becomes more important (e.g. for diagnostic testing), a cutoff value of ≥ 100% may even be more interesting. The lower specificity of the ID-screen could be caused by cross-reactivity with other *Mycoplasma* species, as mixed infections are often present [17, 22]. To better understand the value of the ID-screen in the determination of *M. bovis* herd status, more research should be conducted on the longevity of antibody detection in BTM after *M. bovis* exposure and the influence of (sub)clinically infected animals. For example, use of an in-house antibody ELISA test with a similarly high sensitivity and specificity showed the presence of antibodies for at least 1.5 year without detection of *M. bovis* antigen in the herd [12].

The second test we evaluated was the Bio K432. We observed a very low sensitivity (25.6%), but a high specificity (96.8%). The low sensitivity was not surprisingly since the conjugate (monoclonal a-IgG2 peroxidase) may predispose for a IgG2 production in contrast to the other tests with protein G (Bio K302 and ID screen®) and IgG2 levels are much lower in milk in comparison to IgG1 [23]. It has even been described that in some cattle IgG2 can be absent (e.g. Red Danish Milk Breed) [24]. Nevertheless, IgG2 in milk could increase during inflammation [25], but as milk from mastitis cows is often withheld from the BTM, this test may be more useful as diagnostic test for individual milk samples or to distinguish between calves with and without maternal immunity while testing serum samples [17]. If one insists on using this ELISA for BTM, it seems advisable to adjust the cutoff value to ≤ 20%, to improve sensitivity (82.0%) and specificity (75.1%).

The third test under evaluation, the Bio K302, showed a moderate sensitivity (69.2%) and rather high specificity (85.8%) in our study population. The sensitivity of the Bio K302 was in line with a previous study on BTM from Danish herds (60.4%) [16], though the specificity in our study was a bit lower (97.3%). This could be due to different latent classes, as in previous study the comparison was made between an antibody test and PCR [16]. Another reason could be due to the circulation of other *Mycoplasma* strains and species, as was opted for the reason for the inferior diagnostic performance of this test on serum from Australian cattle [11, 26]. Nielsen et al. (2015) also proposed to adjust cutoff values, but from ≥ 37% to ≥ 50% to improve specificity. This indeed improved the specificity (99.6%), but drastically decreased the sensitivity (43.5%), somewhat in line with our results (59.6% sensitivity, 88.1% specificity). When the aim is to use this ELISA as a screening test, a better diagnostic performance would be obtained when reducing the cutoff to ≤ 30% (76.7% sensitivity, 77.5% specificity). In this case, the final model showed a sensitivity of 85.5% and a specificity of 77.2%. Advantages of the Bio K302 are the knowledge about antibody presence after clinical mastitis (declines after approximately 8 months), number of antibody producing animals who are contributing to the BTM (at least 30% of the herd in case of cutoff ≤ 30%), positive correlation between prevalence and BTM S/P%, and the observation that herds can become negative after a certain amount of time [11, 27, 28]. Therefore, this test is applicable to observe disease spread geographically and change over time in BTM.

Finally, when comparing the apparent prevalence for different tests, a huge difference was observed between the used antibody ELISAs (9.6%, 30.8%, and 50.6%). A true prevalence of 30.8–44.1% was detected for both countries combined when using the manufacturer cutoff values, whereas after adjusting the cutoff values a true prevalence of 26.1% was observed. It is however important to emphasize that our study was based on a convenience sample and not a random sample. Therefore, veterinarians may have targeted herds which were suspected of a *M. bovis* infection rather than those who were not, as a consequence the prevalence as stated by the BLCM cannot be adopted as the true prevalence. The great difference between apparent and true prevalence was also observed on individual serum samples from Dutch herds when using BLCA [19]. Here, a true herd prevalence over 415 herds was estimated at 69.9%, while using the Bio K260 (sensitivity 14.1% and specificity 97.2%). McAloon et al. (2019) [29] showed that BLCA tends to overestimate herd-level true prevalence in case of poor diagnostic test sensitivity. We also observed that the sensitivity analysis of the BLCA showed a great influence on true prevalence. Therefore, next to our sampling bias, BLCA may not be the best method to determine true prevalence, and the results of this study show the importance of knowledge and harmonization of tests and analysis when calculating or comparing prevalence data.

In general, discrepancies in diagnostic performance of tests and studies can be attributed to variations in the population under examination, such as specific antibody responses (e.g. age, breed, clinical status), herd size, milk yield, calving period, seasonal changes, but also to specific test attributes which may render them more susceptible to certain *M. bovis* strains or cross-reactivity with other antigens [11, 15, 30–36]. In our study, we combined BTM samples from Belgian and Swiss herds, which makes it very likely that other *M. bovis* strains and antigens were present in herds [37, 38]. Further exploration in different populations may be necessary, and it is advisable to perform cross-reactivity tests on *M. bovis* antibody ELISA’s with non-*M. bovis* antigens [39].

In conclusion, the benefit from a single determination of antibodies in BTM to assess the *M. bovis* herd status is questionable, as for the moment it does not provide information on the active infectious state of the herd. None of the antibody ELISA tests are perfect, and other studies showed the possibility of antibody negative BTM samples, while PCR on BTM or among samples from calves were positive [6, 12, 20]. Therefore, BTM may be useful for initial screening/monitoring of *M. bovis*, but additional testing (e.g. individual samples, other diagnostic methods, repeated BTM analyses) to determine definitive herd status and before any high impact decision on farm will be made, is highly recommended. Finally, when animals are purchased from BTM-negative herds, additional testing of individual animals remains strongly advisable to reduce the risk of introducing *M. bovis* into a negative herd.

## Methods

### Study population and sampling

A prospective diagnostic test accuracy study on BTM samples from Belgian and Swiss dairy cattle herds was performed. To detect a difference in sensitivity and specificity of 20% (power 80%), the minimum sample size required for a screening study is 103 [40]. Veterinarians were asked to collect BTM samples from herds of which they were interested in the *M. bovis* herd status. There were no criteria on herd size or current herd status, except that samples should be taken directly from the milk cooling tank. We conveniently collected 156 BTM samples from 155 different dairy farms between June 2021 and October 2022. Of these herds 71 were located in Switzerland (mainly eastern and central cantons), and 84 in Belgium (mainly Flanders). One herd submitted two samples, one from the tank milk for diseased animals and one from the tank milk for human consumption. Since no other recent diagnostic results were made known to the investigators, all herds were labelled as ‘herd status unknown’. The BTM samples were taken by collecting 50–100 mL from the tank milk and collected in a collection tube or jar without any preservative. All milk samples were stored at -20 °C (maximum 6 months) before analysis.

### Antibody ELISA tests and interpretation

After thawing, all samples were analyzed blindly (no clinical information available to performer) with three indirect commercially available *M. bovis* ELISA kits: Bio K432 (Bio-X Diagnostics, Rochefort, Belgium), Bio K302 (Bio-X Diagnostics, Rochefort, Belgium), and ID-Screen® *Mycoplasma bovis* (ID Vet, Grabels, France) following manufacturer descriptions for BTM. For the ID-screen the overnight protocol was used. The sample-to-positive percentage (S/P%) for each sample and test was calculated as follows:

$${\text{S}}/{\text{P}}\% = (\frac{{({\text{OD}}sample - {\text{OD}}mean\,negative\,control)}}{{({\text{OD}}mean\,positive\,control - {\text{OD}}mean\,negative\,control)}}) \times 100$$

To determine whether samples were positive or negative the cutoff values recommended by the manufacturer were used. First, BTM samples were labeled positive when the S/P% was ≥ 37% using Bio K302, ≥ 40% using Bio K432, or ≥ 30% while using the ID-screen. However, as also described by the manual, the results of the ID-screen can be semi-quantified, categorizing results in ‘+’ (S/P% ≥30%), ‘++’ (≥ 50%), ‘+++’ (≥ 100%), or ‘++++’ (≥ 150%). Therefore, secondly, results of the ID-screen were categorized following these cutoff values. Finally, as the sensitivity of Bio-X tests is often low [14, 16, 30], results of the Bio K302 and Bio K432 were categorized by invented cutoff values with decreasing intervals of 7–10% (range 50 − 10%) to explore whether there are more optimal S/P% cutoff values for a higher diagnostic test accuracy. By decreasing the cutoff values, we would expect an increase in sensitivity, and therefore less false negative BTM samples.

### Bayesian latent class models

#### Model development

A gold standard for *M. bovis* antibody testing is lacking, therefore a Bayesian latent class analysis (BLCA) was performed to determine the diagnostic accuracy of the tested antibody ELISAs following the same protocol as described before [17]. In brief, both an independent (all diagnostic tests are considered to be equal) and conditional dependent model (two tests are considered to be more similar in comparison to the third) were built to compare the Bio K302, Bio K432, and ID-screen antibody ELISA tests. Models and model fit were evaluated by visual comparison and deviance information criterion (DIC), respectively. When the difference between models is less than three, models are considered not to be statistically different [41]. Two codes (one for the conditional dependent and one for the independent model) kindly provided by Dr. S. Buczinski (University of Montreal, Montreal, Canada) [42, 43] were used to determine the diagnostic test accuracy (sensitivity and specificity) of the three antibody ELISAs (ID Screen, Bio K302, Bio K432), and the prevalence of herds with *M. bovis* antibodies in this study population.

#### Prior distribution determination

Prior information was obtained from previous publications and added as informative priors. For the diagnostic test accuracy of Bio K302 results from Nielsen et al. (2015) were used, being a sensitivity of 60.4% and specificity of 97.3% at a cutoff of ≥ 37%. For the prevalence, an estimate of the average in both Belgium and Switzerland was used, based on different studies [6, 44, 45], which resulted in a prevalence of 40%. Priors consist of probability distributions around a specified value, which we derived from Epitools (Ausvet Animal Health Services, https://epitools.ausvet.com.au/betaparamsone) by including a 95th percentile of 37%, 94%, and 16% for Bio K302 sensitivity, Bio K302 specificity, and prevalence in Belgium and Switzerland, respectively [6, 16, 44, 45]. This resulted in the following beta distributions: Beta(8.086, 5.646) and Beta(147.175, 5.0562) for Bio K302 (sensitivity and specificity), and Beta(3.223, 4.335) for prevalence. For the covariance in *M. bovis* negative animals (covDn) and *M. bovis* positive animals (covDp) universals were used.

#### Model analysis

First, three models were run for the conditional independent and dependent tests in WinBUGS statistical freeware version 1.4.3. (MRC Biostatistics Unit, Cambridge, UK) using Gibbs sampling as previously described [17]. If case models did not comply due to extreme values, automatic generation of chains was used. In the first model all prior information was set at uninformative (Beta 1,1), in the second model prevalence was included and in the third model also sensitivity and specificity of the Bio K302 were added. Secondly, four independent models without prior information were run for ID-screen S/P% cutoff ≥ 30% (CO_≥ 30%_), 50% (CO_≥ 50%_), 100% (CO_≥ 100%_) and 150% (CO_≥ 150%_) compared to Bio K302 and Bio K342, to determine the optimal cutoff value for the ID-screen. Third, models without prior information were run for a range of different S/P% cutoff values for Bio K302 (20–50%) and Bio K342 (10–50%). The optimal cutoff value for the diagnostic test was determined by the results of the BLCA and the highest Youden’s Index (sensitivity + specificity − 1) [46]. Finally, the model with the optimal S/P% cutoff value for every test was run. To determine the robustness of the first and final model, an extensive sensitivity analysis was performed for the initial and final model containing the three antibody ELISA tests. Alternative models with very different prior specifications than the main model were run and inspected whether posterior estimates of these alternative models were included in the 95% CI of the main model.

### Electronic supplementary material

Below is the link to the electronic supplementary material.

Supplementary Material 1

## Data Availability

Data is available on request from the authors.

## Abbreviations

BLCA: Bayesian latent class analysis
BTM: Bulk tank milk
CI: Credible interval
CO: Cutoff
ELISA: Enzyme-linked immunosorbent assay
*M. bovis*: *Mycoplasmopsis bovis/Mycoplasma bovis*
PCR: Polymerase chain reaction
Se: Sensitivity
Sp: Specificity
S/P%: Sample-to-positive percentage
