# Supplementary material for: Diagnostic performance of Mycoplasmopsis bovis antibody ELISA tests on bulk tank milk from dairy herds
Source: BMC Vet Res. 2024 Mar 5;20:81. doi: 10.1186/s12917-024-03927-x (PMC10916218; doi:10.1186/s12917-024-03927-x)
Supplement: Supplementary file 1 — Supplementary Material 1 [file 12917_2024_3927_MOESM1_ESM.pdf]

Supplementary file 1. Overview of result (positive = 1, negative = 0) per herd, test (ID = ID-screen, K432 = Bio K432, K302 = Bio K302) and _cutoff value.

BTM no	*ID_30*	*ID_50*	*ID_100*	*ID_150*	*K302_10*	*K302_20*	*K302_30*	*K302_37*	*K302_40*	*K302_50*	*K432_10*	*K432_20*	*K432_30*	*K432_40*	*K432_50*
1	1	1	1	0	1	1	1	1	1	1	1	1	1	1	1
2	1	0	0	0	1	1	0	0	0	0	1	1	1	0	0
3	0	0	0	0	1	1	0	0	0	0	1	1	0	0	0
4	0	0	0	0	1	0	0	0	0	0	1	1	0	0	0
5	0	0	0	0	1	0	0	0	0	0	0	0	0	0	0
6	0	0	0	0	1	0	0	0	0	0	0	0	0	0	0
7	0	0	0	0	1	1	0	0	0	0	1	1	0	0	0
8	0	0	0	0	1	0	0	0	0	0	1	0	0	0	0
9	0	0	0	0	1	0	0	0	0	0	1	0	0	0	0
10	0	0	0	0	1	1	0	0	0	0	1	0	0	0	0
11	1	1	0	0	1	1	1	1	1	1	1	1	0	0	0
12	0	0	0	0	1	1	0	0	0	0	1	0	0	0	0
13	0	0	0	0	1	1	0	0	0	0	1	0	0	0	0
14	0	0	0	0	1	1	1	0	0	0	1	1	0	0	0
15	1	1	0	0	1	1	1	0	0	0	1	1	0	0	0
16	0	0	0	0	1	1	0	0	0	0	1	1	0	0	0
17	1	1	1	0	1	1	1	1	1	1	1	1	1	0	0
18	1	0	0	0	1	1	1	1	1	1	1	1	0	0	0
19	1	0	0	0	1	1	1	1	0	0	1	1	1	0	0
20	1	1	0	0	1	1	1	1	1	0	1	0	0	0	0
21	1	1	1	0	1	1	1	1	1	0	1	1	0	0	0
22	1	1	0	0	1	1	1	0	0	0	1	1	0	0	0
23	1	1	1	0	1	1	1	1	1	1	1	0	0	0	0
24	1	0	0	0	1	1	1	1	0	0	1	1	0	0	0
25	1	1	0	0	1	1	1	0	0	0	1	1	1	1	0
26	1	0	0	0	1	1	0	0	0	0	1	1	1	0	0
27	0	0	0	0	1	1	0	0	0	0	1	0	0	0	0
28	1	1	1	0	1	1	1	1	1	1	1	1	1	0	0
29	1	1	0	0	1	1	1	1	1	1	1	1	0	0	0
30	1	1	1	0	1	1	1	1	1	1	1	1	0	0	0
31	0	0	0	0	1	1	0	0	0	0	1	1	0	0	0

32	0	0	0	0	1	1	0	0	0	0	1	1	1	0	0
33	0	0	0	0	0	0	0	0	0	0	0	0	0	0	0
34	0	0	0	0	1	1	0	0	0	0	1	0	0	0	0
35	1	1	1	0	1	1	1	1	1	1	1	1	1	1	1
36	1	1	1	1	1	1	1	1	1	1	1	1	1	1	1
37	0	0	0	0	1	1	0	0	0	0	1	0	0	0	0
38	1	1	1	0	1	1	1	1	1	1	1	1	1	1	1
39	0	0	0	0	1	0	0	0	0	0	0	0	0	0	0
40	0	0	0	0	1	0	0	0	0	0	0	0	0	0	0
41	0	0	0	0	1	0	0	0	0	0	1	0	0	0	0
42	0	0	0	0	1	0	0	0	0	0	1	0	0	0	0
43	0	0	0	0	1	1	0	0	0	0	1	1	0	0	0
44	1	1	1	0	1	1	1	1	1	1	1	1	1	1	1
45	1	1	0	0	1	1	1	1	1	1	1	1	1	0	0
46	1	1	1	0	1	1	1	1	1	1	1	1	1	0	0
47	1	0	0	0	1	1	0	0	0	0	1	1	1	0	0
48	0	0	0	0	1	1	1	0	0	0	1	1	1	1	0
49	0	0	0	0	1	0	0	0	0	0	1	1	0	0	0
50	1	1	0	0	1	1	1	1	1	1	1	1	1	1	0
51	1	0	0	0	1	1	1	1	1	1	1	0	0	0	0
52	0	0	0	0	1	1	0	0	0	0	1	1	0	0	0
53	1	0	0	0	1	1	1	1	1	0	1	1	0	0	0
54	1	0	0	0	1	1	1	1	1	0	1	1	0	0	0
55	1	1	0	0	1	1	1	1	1	0	1	1	1	0	0
56	1	1	0	0	1	1	1	0	0	0	1	1	0	0	0
57	0	0	0	0	1	1	0	0	0	0	1	1	1	1	0
58	1	1	0	0	1	1	0	0	0	0	1	1	0	0	0
59	0	0	0	0	1	1	0	0	0	0	1	0	0	0	0
60	1	1	1	0	1	1	1	1	1	1	1	1	0	0	0
61	1	0	0	0	1	1	1	1	1	0	1	0	0	0	0
62	1	1	0	0	1	1	1	1	1	0	1	1	1	1	0
63	1	1	1	1	1	1	1	1	1	1	1	1	1	1	1
64	1	1	1	1	1	1	1	1	1	1	1	1	1	1	1

65	1	1	0	0	1	1	1	1	1	1	1	1	0	0	0
66	0	0	0	0	1	1	0	0	0	0	0	0	0	0	0
67	0	0	0	0	1	1	0	0	0	0	1	0	0	0	0
68	1	0	0	0	1	1	0	0	0	0	0	0	0	0	0
69	1	0	0	0	1	1	0	0	0	0	1	0	0	0	0
70	0	0	0	0	1	0	0	0	0	0	0	0	0	0	0
71	0	0	0	0	0	0	0	0	0	0	1	0	0	0	0
72	1	0	0	0	0	0	0	0	0	0	1	0	0	0	0
73	0	0	0	0	1	0	0	0	0	0	1	0	0	0	0
74	0	0	0	0	1	0	0	0	0	0	1	0	0	0	0
75	1	1	0	0	1	1	0	0	0	0	1	0	0	0	0
76	0	0	0	0	1	1	0	0	0	0	1	0	0	0	0
77	0	0	0	0	1	1	0	0	0	0	0	0	0	0	0
78	0	0	0	0	1	0	0	0	0	0	1	1	0	0	0
79	0	0	0	0	1	0	0	0	0	0	1	0	0	0	0
80	0	0	0	0	1	0	0	0	0	0	0	0	0	0	0
81	1	0	0	0	1	0	0	0	0	0	0	0	0	0	0
82	1	0	0	0	1	1	0	0	0	0	1	1	0	0	0
83	0	0	0	0	1	0	0	0	0	0	0	0	0	0	0
84	0	0	0	0	1	1	0	0	0	0	1	0	0	0	0
85	0	0	0	0	1	1	1	1	0	0	1	0	0	0	0
86	0	0	0	0	1	1	0	0	0	0	0	0	0	0	0
87	0	0	0	0	1	1	0	0	0	0	1	0	0	0	0
88	1	1	0	0	1	0	0	0	0	0	1	0	0	0	0
89	0	0	0	0	1	1	0	0	0	0	1	0	0	0	0
90	0	0	0	0	1	0	0	0	0	0	1	0	0	0	0
91	1	0	0	0	0	0	0	0	0	0	1	0	0	0	0
92	0	0	0	0	1	0	0	0	0	0	1	0	0	0	0
93	1	0	0	0	1	1	0	0	0	0	1	1	0	0	0
94	0	0	0	0	1	1	1	1	1	1	1	0	0	0	0
95	1	0	0	0	1	1	1	1	1	0	1	0	0	0	0
96	0	0	0	0	1	1	1	1	1	1	0	0	0	0	0
97	0	0	0	0	1	1	1	1	1	1	0	0	0	0	0

98	0	0	0	0	1	0	0	0	0	0	0	0	0	0	0
99	0	0	0	0	1	0	0	0	0	0	1	0	0	0	0
100	0	0	0	0	1	1	1	1	1	1	0	0	0	0	0
101	1	1	1	0	0	0	0	0	0	0	1	1	0	0	0
102	1	1	1	0	1	0	0	0	0	0	1	1	1	1	1
103	1	1	1	0	1	1	0	0	0	0	1	1	1	1	0
104	0	0	0	0	1	0	0	0	0	0	0	0	0	0	0
105	0	0	0	0	1	0	0	0	0	0	1	0	0	0	0
106	1	1	1	1	1	1	1	0	0	0	1	1	1	0	0
107	0	0	0	0	1	1	1	0	0	0	0	0	0	0	0
108	0	0	0	0	1	0	0	0	0	0	0	0	0	0	0
109	0	0	0	0	1	1	1	1	1	1	0	0	0	0	0
110	1	0	0	0	1	1	0	0	0	0	1	0	0	0	0
111	1	0	0	0	1	1	0	0	0	0	1	0	0	0	0
112	1	0	0	0	1	1	0	0	0	0	1	0	0	0	0
113	1	0	0	0	1	1	1	1	1	1	1	0	0	0	0
114	0	0	0	0	1	1	1	1	1	1	1	0	0	0	0
115	1	1	0	0	1	0	0	0	0	0	1	0	0	0	0
116	1	0	0	0	0	0	0	0	0	0	1	1	0	0	0
117	1	1	0	0	1	1	0	0	0	0	1	1	0	0	0
118	1	0	0	0	1	0	0	0	0	0	1	1	0	0	0
119	1	1	1	1	1	1	1	1	1	1	1	1	0	0	0
120	1	1	1	1	1	1	1	1	1	1	1	1	1	0	0
121	1	1	0	0	1	1	1	0	0	0	1	0	0	0	0
122	0	0	0	0	0	0	0	0	0	0	0	0	0	0	0
123	1	1	1	1	1	0	0	0	0	0	1	1	1	1	1
124	0	0	0	0	1	0	0	0	0	0	1	0	0	0	0
125	1	1	0	0	0	0	0	0	0	0	1	1	0	0	0
126	0	0	0	0	0	0	0	0	0	0	0	0	0	0	0
127	0	0	0	0	0	0	0	0	0	0	0	0	0	0	0
128	0	0	0	0	0	0	0	0	0	0	0	0	0	0	0
129	0	0	0	0	0	0	0	0	0	0	0	0	0	0	0
130	0	0	0	0	0	0	0	0	0	0	0	0	0	0	0

131	0	0	0	0	1	1	1	1	1	1	0	0	0	0	0
132	0	0	0	0	1	1	1	1	1	1	0	0	0	0	0
133	0	0	0	0	1	1	1	1	1	0	0	0	0	0	0
134	0	0	0	0	1	1	1	1	1	0	0	0	0	0	0
135	0	0	0	0	1	1	0	0	0	0	0	0	0	0	0
136	0	0	0	0	1	1	0	0	0	0	0	0	0	0	0
137	1	1	1	1	1	1	0	0	0	0	1	0	0	0	0
138	1	1	1	0	1	0	0	0	0	0	1	0	0	0	0
139	1	1	1	0	1	1	0	0	0	0	0	0	0	0	0
140	1	1	1	0	1	1	1	0	0	0	1	0	0	0	0
141	1	1	0	0	1	1	1	0	0	0	1	0	0	0	0
142	0	0	0	0	1	1	1	0	0	0	0	0	0	0	0
143	1	1	0	0	1	1	1	1	1	0	1	0	0	0	0
144	0	0	0	0	1	1	1	1	1	1	0	0	0	0	0
145	1	1	0	0	1	1	1	0	0	0	1	0	0	0	0
146	1	1	0	0	1	0	0	0	0	0	0	0	0	0	0
147	1	1	0	0	1	1	0	0	0	0	0	0	0	0	0
148	1	1	0	0	1	0	0	0	0	0	0	0	0	0	0
149	1	1	1	0	1	0	0	0	0	0	0	0	0	0	0
150	1	1	1	0	1	1	0	0	0	0	1	0	0	0	0
151	1	1	0	0	1	1	1	1	1	0	0	0	0	0	0
152	1	1	1	0	1	1	0	0	0	0	1	0	0	0	0
153	1	1	1	1	1	1	1	1	1	1	1	1	0	0	0
154	0	0	0	0	1	0	0	0	0	0	1	0	0	0	0
155	0	0	0	0	1	0	0	0	0	0	1	1	0	0	0
156	0	0	0	0	1	0	0	0	0	0	1	1	0	0	0
% positive	50,64	35,26	18,59	5,77	91,67	67,31	39,10	30,77	28,85	21,15	73,72	39,74	17,31	9,62	5,77
